# Contig-Layout-Authenticator (CLA): A Combinatorial Approach to Ordering and Scaffolding of Bacterial Contigs for Comparative Genomics and Molecular Epidemiology

**DOI:** 10.1371/journal.pone.0155459

**Published:** 2016-06-01

**Authors:** Sabiha Shaik, Narender Kumar, Aditya K. Lankapalli, Sumeet K. Tiwari, Ramani Baddam, Niyaz Ahmed

**Affiliations:** Pathogen Biology Laboratory, Department of Biotechnology and Bioinformatics, School of Life Sciences, University of Hyderabad, Hyderabad, India; Massey University, NEW ZEALAND

## Abstract

A wide variety of genome sequencing platforms have emerged in the recent past. High-throughput platforms like Illumina and 454 are essentially adaptations of the shotgun approach generating millions of fragmented single or paired sequencing reads. To reconstruct whole genomes, the reads have to be assembled into contigs, which often require further downstream processing. The contigs can be directly ordered according to a reference, scaffolded based on paired read information, or assembled using a combination of the two approaches. While the reference-based approach appears to mask strain-specific information, scaffolding based on paired-end information suffers when repetitive elements longer than the size of the sequencing reads are present in the genome. Sequencing technologies that produce long reads can solve the problems associated with repetitive elements but are not necessarily easily available to researchers. The most common high-throughput technology currently used is the Illumina short read platform. To improve upon the shortcomings associated with the construction of draft genomes with Illumina paired-end sequencing, we developed Contig-Layout-Authenticator (CLA). The CLA pipeline can scaffold reference-sorted contigs based on paired reads, resulting in better assembled genomes. Moreover, CLA also hints at probable misassemblies and contaminations, for the users to cross-check before constructing the consensus draft. The CLA pipeline was designed and trained extensively on various bacterial genome datasets for the ordering and scaffolding of large repetitive contigs. The tool has been validated and compared favorably with other widely-used scaffolding and ordering tools using both simulated and real sequence datasets. CLA is a user friendly tool that requires a single command line input to generate ordered scaffolds.

## Introduction

The emergence of newer platforms for whole genome sequencing has driven a revolution in the field of comparative genomics and epidemiological tracking of microorganisms [1–3]. Some of these platforms are essentially an adaptation of shotgun sequencing producing higher throughput and being cost-effective when compared to traditional Sanger sequencing. Though there are a variety of platforms available, such as Illumina, 454, Ion Torrent etc., their utility mainly entails reading of bases from short DNA fragments to generate read data [4]. In addition, some platforms such as Illumina also provide a paired-end sequencing option which essentially means that the generated fragments are read from both the ends providing useful information for downstream processing. Although the advent of these platforms has revolutionized the way we analyze and compare genomes, the main challenge refers to reconstructing the complete or a maximum draft genome out of millions of reads generated by the sequencers. This has underpinned development of multiple assembly tools with similar or distinct algorithms to harness and consolidate the sequence read data into larger contiguous fragments called contigs. Some examples of such assembly tools based on *de novo* algorithms include Velvet [5], SSAKE [6], etc. Although many of the available assembly processes reduce complexity of the data by generating contigs, the latter still require a lot of downstream analysis such as sorting based on their real biological order and resolution of repetitive elements before a high quality draft or complete genome is constructed.

The assembled contigs could be incorporated in various ways to reconstruct the genome. One important approach refers to directly ordering the contigs according to a chosen reference to build a chromosomal assembly with the help of tools such as ABACAS [7], Ragout [8], Mauve Contig Mover (MCM) [9], CONTIGuator [10] and Scaffold_builder [11] etc. These reference based approaches are known to be very efficient in analyzing specific mutations/variations among highly similar organisms since the possibilities of finding novel genomic insertions are scarce in some clonal populations. However, these methods have limitations in identifying strain-specific genes as well as handling mobile genetic elements which usually contribute to high strain to strain variation [12]. These approaches could therefore affect the resolution of an assembled genome sequence, especially in case of highly diverse and recombining organisms such as *Helicobacter pylori* [13].

The other approach for genome assembly is based on constructing scaffolds consisting of contigs joined together based on paired-end read information [14]. While a majority of sequence read assemblers such as Velvet [5], SOAPdenovo2 [15], SOPRA [16] and SGA [17] etc. come with an additional option of scaffolding, exclusive and stand-alone scaffolding tools such as Bambus [14], Bambus2 [18] and SSPACE [19] are also available. These tools usually generate multiple scaffolds where only intra-scaffold ordering and orientation of contigs is observed. Therefore, further ordering of scaffolds is required to obtain a draft genome. Other than this, these tools also face difficulties during the scaffolding of the repetitive contigs which sometimes result in misjoining/misassembly of the scaffolds [20]. The emergence of sequencing platforms that no longer depend on genome fragmentation and attempt to read directly from the genomic DNA such as PacBio, Oxford Nanopore etc. has shown promise to overcome these limitations by virtue of the long reads they generate. However, these platforms would take some more time to become mainstream and to displace some of the popular yet affordable platforms such as Illumina in terms of availability and affordability in some parts of the world [21–23].

In order to address the limitations of both the reference and scaffolding based whole genome ordering tools mainly entailing Illumina paired-end sequencing, we developed Contig-Layout-Authenticator (CLA) which not only scaffolds the contigs but also attempts to achieve proper ordering of the scaffolds at the whole genome level. In other words, the contigs are initially ordered at the whole genome level similar to the ordering achieved by ABACAS and Ragout, based on a reference, but the order is much more refined at the later stages. The ordered contigs are further scaffolded when supported by the paired-end information; this way, if multiple scaffolds are generated, then it clearly indicates that some genome information between two consecutive scaffolds still needs to be incorporated into the assembly. Thus, CLA carries out the functions of ordering and scaffolding tools in a combinatorial and efficient manner yielding better draft genomes. The pipeline of CLA is proficient and adept in identifying probable contaminating contigs as well as misassemblies within the scaffolds. It assists the users in improving quality of the draft genome in an informed and step by step manner. The comparative analysis of CLA with other widely used ordering and scaffolding tools revealed its enhanced performance. Therefore, we believe CLA would serve as an efficient and user friendly tool for sorting and scaffolding of contigs to achieve better draft genomes of prokaryotic organisms.

## Results

As CLA performs both ordering at the whole genome level followed by scaffolding, its efficiency was validated at three different levels. Firstly, it was compared with the widely used ordering tools such as ABACAS, MCM, CONTIGuator and Ragout. Secondly, the scaffolding ability of CLA was compared with Bambus2, SSPACE, SOPRA, SOAPdenovo2, SGA and the reference-based scaffolder, MeDuSa [24]. Finally, to test the hybrid approach efficiency, scaffolds from SSPACE, SOPRA, SOAPdenovo2 and SGA were further ordered with Ragout and the final results were compared against CLA. All the in-house scripts (along with their command line history) that were used for validation, are provided along with the tool.

### CLA versus reference based sorting tools

All the tools under this category such as ABACAS (v1.3.1), MCM (Mauve-2.3.1), CONTIGuator (v2.7.3) and Ragout (v1.1) were run with default parameters and their outputs were compared with CLA using the QUAST(v3.1) [25] tool to avoid manual errors. Ragout and MeDuSa were run using multiple reference genomes (S1 and S2 Tables). The outputs generated by all the above mentioned tools using simulated and real sequence datasets were evaluated for parameters such as misassemblies within the chromosome assembly, the number of contigs (>1kb) unassigned in the final order, the size of largest unassigned contig, the genome fraction obtained and the number of large repetitive contigs (contig size greater than 500bp) correctly placed. QUAST was used to identify number of misassemblies and to calculate the genome fractions with the original genome as reference for simulated datasets. While in the case of a real dataset, due to the unavailability of the original genome, a closely related genome was used as reference for QUAST. Therefore, the statistics generated in case of real dataset could be biased towards the reference genome and may not exactly depict the efficiency of the tools as shown with the simulated dataset. In-house Perl scripts were used to calculate the number of large repetitive contigs present in the original genome, as well as number of them placed by each of these tools.

### Misassemblies in the chromosome assembly

A chromosomal assembly or draft genome constructed from reference based ordering tools comprised of ordered contigs merged together after placing ‘Ns’ in between. Tools such as Ragout, CONTIGuator and ABACAS usually provide the final ordered contigs in the form of a single scaffold or chromosome assembly, whereas, MCM only provides ordered contigs in multi-FASTA format. In CLA, contigs are merged into scaffolds (by placing ‘Ns’ in between) only if supported by sufficient paired-end information. An in-house script was used to generate a chromosome assembly for the outputs of MCM and CLA. In order to identify the errors such as misassemblies encountered by each of the compared tools and judge their performance, we used QUAST. The misassemblies observed in the chromosomal assemblies were further linked to inversions, relocations and translocations by QUAST. Information about different types of misassemblies observed in case of simulated dataset(s) is given in S3 Table. As inferred from Table 1, CLA outcompeted other tools by producing least number of misassemblies in case of simulated datasets of all the test organisms except *H*. *pylori*. In case of organisms such as *Bartonella quintana*, and *Caulobacter crescentus*, CLA showed only two inversions without any relocations (S3 Table). After CLA, Ragout and CONTIGuator performed fairly well followed by ABACAS. MCM was observed to be the only tool with the highest number of misplacements in all organisms; this might be due to placement of unassigned contigs at the end of the order.

**Table 1:** Comparative statistics of CLA and reference based alignment tools generated using simulated dataset.

	Genome*	Tool	# misassemblies in chromosome assembly	Unassigned contigs in final order >1kb	Largest contig unassigned	Genome fraction	True repeat places filled**
**1.**	***B*. *Quintana*** #contigs:**47** #total repeat positions: **19** #misassemblies in input contigs: **0**	CLA	2	0	850	99.68	15
		Ragout	5	3	3347	99.299	15
		ABACAS	12	1	1104	98.648	9
		MCM	26	0	0	98.767	9
		CONTIGuator	4	7	10343	97.401	0
**2.**	***C*. *jejuni*** #contigs: **33** #total repeat positions: **12** #misassemblies in input contigs: **0**	CLA	4	0	425	99.728	10
		Ragout	5	4	4566	98.807	10
		ABACAS	9	7	481308	56.49	3
		MCM	21	0	0	98.82	5
		CONTIGuator	8	6	6071	97.71	1
**3.**	***C*. *crescentus*** #contigs:**49** #total repeat positions: **28** #misassemblies in input contigs: **0**	CLA	2	0	793	99.901	24
		Ragout	8	0	371	99.923	28
		ABACAS	11	0	371	99.355	9
		MCM	18	0	0	99.355	9
		CONTIGuator	4	6	5440	98.94	0
**4.**	***H*. *influenzae*** #contigs: **43** #total repeat positions: **19** #misassemblies in input contigs: **1**	CLA	6	0	606	98.963	9
		Ragout	12	0	375	98.559	19
		ABACAS	15	4	345463	70.201	3
		MCM	22	0	0	98.187	5
		CONTIGuator	11	3	3503	97.719	1
**5.**	***H*. *pylori*** #contigs: **46** #total repeat positions: **20** #misassemblies in input contigs: **0**	CLA	7	0	960	99.672	17
		Ragout	4	1	5487	98.97	14
		ABACAS	17	6	170928	82.357	6
		MCM	23	0	0	99.133	10
		CONTIGuator	8	4	4051	98.135	0
**6.**	***R*. *etli*** #contigs: **30** #total repeat positions: **21** #misassemblies in input contigs: **0**	CLA	3	0	601	99.756	19
		Ragout	5	1	1052	99.406	13
		ABACAS	5	7	798023	71.125	4
		MCM	24	0	0	99.221	8
		CONTIGuator	10	8	4331	98.761	0
**7.**	***S*. Typhi** #contigs: **67** #total repeat positions: **24** #misassemblies in input contigs: **0**	CLA	8	0	520	99.141	11
		Ragout	55	2	3835	98.873	12
		ABACAS	13	0	474	98.682	7
		MCM	32	0	0	98.698	7
		CONTIGuator	13	5	6299	98.311	1
**8.**	***T*. *pallidum*** #contigs: **22** #total repeat positions: **17** #misassemblies in input contigs: **0**	CLA	5	0	980	99.124	9
		Ragout	7	1	1617	99.432	17
		ABACAS	10	0	0	98.768	7
		MCM	15	0	0	98.769	7
		CONTIGuator	6	3	3283	97.95	1

*All the genomes were simulated with a read length of 100bp and insert size of 400bp.

** Total number of positions filled by the repetitive contigs in the final output; for example, if tools have placed contig A at 2 out of 4 places and contig B at 1 out of 6 places, then total number of repeat positions in the original genome would be 10 and true repeats placed by the tool would be 3.

^#^ Number of

While dealing with real datasets, a reference genome required to act as an exact representative of the test strains was not available for some bacterial species/strains; we therefore chose a closely related genome as reference. Because this modification would definitely favor the reference based tools, the performance of CLA varied from genome to genome as revealed in the Table 2. CLA vended least number of misassemblies in all cases except *Campylobacter jejuni*, as inferred from Table 2. Moreover, the performance of CLA was comparable and even superior in some instances to other tools thus exhibiting the robustness of the combinatorial approach it used.

**Table 2:** Comparative statistics of CLA and reference based alignment tools generated using real dataset.

	Genome	Tool	# misassemblies in chromosome assembly	Unassigned contigs in final order >1kb	Largest contig unassigned	Genome fraction	True repeat places filled**
**1.**	***C*. *jejuni*** #contigs: **72** Read length: **100** Insert: **300** #repeats: **11** #misassemblies in input contigs: **7**	CLA	19	0	965	90.553	3
		Ragout	18	8	32459	91.261	11
		ABACAS	22	8	32459	90.584	4
		MCM	39	0	0	90.76	4
		CONTIGuator	20	8	32459	90.051	0
**2.**	***E*. *coli*** #contigs: **252** Read length: **151** Insert: **300** #repeats: **51** #misassemblies in input contigs: **98**	CLA	231	0	1000	92.245	15
		Ragout	242	45	36453	91.65	36
		ABACAS	203	49	36453	91.264	10
		MCM	262	0	0	92.054	10
		CONTIGuator	176	46	36453	91.508	0
**3.**	***H*. *influenzae*** #contigs: **50** Read length: **100** Insert: **200** #repeats: **19** #misassemblies in input contigs: **25**	CLA	32	0	774	88.984	8
		Ragout	33	8	5468	89.293	15
		ABACAS	24	11	208372	66.488	4
		MCM	39	0	0	88.194	4
		CONTIGuator	33	10	6440	87.842	0
**4.**	***M*. *tuberculosis*** #contigs: **241** Read length: **101** Insert: **300** #repeats: **43** #misassemblies in input contigs: **6**	CLA	23	0	978	98.491	24
		Ragout	32	9	5385	98.375	38
		ABACAS	37	5	6881	98.441	13
		MCM	113	0	0	98.559	14
		CONTIGuator	24	28	5385	97.11	0
**5.**	***S*. Typhi** #contigs: **119** Read length: **100** Insert: **200** #repeats: **37** #misassemblies in input contigs: **2**	CLA	31	0	978	98.75	13
		Ragout	32	4	93926	99.067	30
		ABACAS	23	4	93926	98.047	11
		MCM	65	0	0	98.812	11
		CONTIGuator	17	10	93926	98.328	0

** Total number of positions filled by the repetitive contigs in the final output.

^#^ Number of

### Contigs unassigned in the final chromosome assembly

Majority of the reference based ordering tools often exclude contigs (which fail to align properly with a reference genome) from the final order. These unassigned contigs are either provided as separate files or put towards the end in their output. The scenario becomes more pertinent in cases of highly recombining organisms as the reference genome architecture might differ significantly from the target genome. These unassigned contigs might encompass valuable strain specific information encoding novel elements and virulence genes etc. Since CLA utilizes paired-end information in addition to a reference genome, it is able to incorporate the maximum number of contigs in the chromosomal assembly and thereby retaining potentially novel, strain specific elements. As observed from the comparative statistics mentioned in Table 1, CLA showed consistent results by not having unassigned any contig with size greater than 1kb in all the organisms. On the other hand, CONTIGuator left more number of contigs with size greater than 1kb unassigned to the final order. It was also observed that a contig as long as 10kb in *B*. *quintana* was not considered in the final order generated by CONTIGuator. Though ABACAS performed considerably well, in some cases such as *C*. *jejuni*, *Haemophilus influenzae*, *H*. *pylori* and *Rhizobium etli*, it ended up excluding contigs of lengths 481kb, 345kb, 170kb and 798kb, respectively, in these organisms. MCM was observed to assign all the contigs in the final assembly as it places the unaligned contigs towards the end of the chromosomal assembly.

A similar pattern was observed with the real dataset (Table 2). While CLA and MCM have both incorporated in the final order all the contigs that were greater than 1kb, contigs as large as 32kb, 36kb and 93kb remained unassigned, respectively, in the final order, when Ragout, ABACAS and CONTIGuator were used to assemble genomes of *C*. *jejuni*, *Escherichia coli* and *Salmonella* Typhi. The number of contigs that were >1kb and were not considered by these tools were also observed to be very high in case of the real dataset.

### Genome Fraction

The genome fraction obtained after the chromosome assembly of all the strains in simulated and real sequence datasets was calculated using QUAST. In almost all cases in simulated datasets, CLA and Ragout were observed to generate a higher genome fraction when compared to others. While the majority of tools achieved a genome fraction greater than 97%, the genome fraction from ABACAS was found to be 56.49%, 70.20%, 82.35% and 71.12% in *C*. *jejuni*, *H*. *influenzae*, *H*. *pylori* and *R*. *etli*, respectively (Table 1). In real datasets, the genome fraction obtained by all the tools was observed to be above 85% except in *H*. *influenzae*, where ABACAS was observed to generate a fraction only equal to 66.48%.

### Resolving large repetitive contigs

Large repetitive contigs were defined as the contigs of length >500 bases having more than one coordinate in the original genome. To validate the placement, large repeat containing contigs of size >500 bases were considered. An in-house script was used to calculate the total number of positions for all repetitive contigs based on a BLASTn [26] alignment against a reference genome. BLAST version 2.2.29+ was used and the best hit with >90% identity and >90% query coverage was considered as a valid hit to identify the position coordinates. Then, the output of CLA and all ordering tools were scanned using another in house Perl script to evaluate the total number of positions filled by repetitive region containing contigs.

The ultimate aim of any genome sequencing project is to harness as much information as possible by formulation of better assemblies. Given this, resolution of repeats becomes an unavoidable parameter to obtain near perfect genomes. Since all the tools mentioned above are designed to aid researchers in achieving better assemblies, these tools were also compared for their performance related to the resolution of repeats. Except CLA and Ragout, other tools under this category were designed only to hint at the probable repetitive contigs and therefore required further manual intervention. As both CLA and Ragout handled repeats to some extent, they performed much better than the other tools in all the genomes. CLA and Ragout could more or less fill a similar number of true repeating positions in the majority of cases. While CLA could correctly fill 19 out of 21 positions (Table 1) in *R*. *etli*, Ragout could fill only 13. Similarly, Ragout performed better in the *Treponema pallidum* (*T*. *pallidum)* assembly by filling all 17 positions whereas only 9 of them could be placed by CLA. Both CLA and Ragout have shown similar performance in highly recombining organisms like *B*. *quintana and C*. *jejuni*, by filling 15 out of 19 positions and 10 out of 12 positions respectively. MCM and CONTIGuator were observed to be placing very few repeats in the correct order and copy number. CLA and Ragout performed in a superior way to other tools in handling the repetitive contigs by placing them in correct positions, followed by ABACAS.

In a real dataset, the number of repetitive positions was calculated with respect to the closely related genome used as reference which also served as input to these tools due to the unavailability of the original genome. Ragout was observed to perform well in all cases followed by CLA as inferred from Table 2. Therefore, it can be surmised that CLA and Ragout are comparable for this parameter in the chromosomal assembly process.

### CLA versus Scaffolding tools

Scaffolding further reduces the number of sequence fragments by joining consecutive contigs and helps in the improvement of assembly. This could be achieved with the help of paired-end information or cues from the assembly graph, but was also attempted with the help of multiple reference genomes in the case of MeDuSa.

CLA, which utilizes a combination of reference alignment and paired-end information for scaffolding was compared against the recently published MeDuSa (v3) and other scaffolders such as SSPACE (v2.0), SOPRA (v1.4.6), SOAP denovo2 (v2.04-r240), SGA and BAMBUS2 (amos-3.1.0). For the simulated dataset, QUAST was used to deduce the assembly statistics from output files of each tool keeping the original genome as reference. Whereas, for the real dataset, genome of a closely related strain was used as reference genome. Parameters such as number of scaffolds formed, number of misassemblies within the scaffolds, number of misassembled scaffolds and size of genome fraction were used to compare the performance of CLA with others.

It was observed that CLA performed better under this category owing to its combinatorial approach. The comparative statistics for simulated and real datasets are mentioned in Tables 3 and 4, respectively. The lesser number of scaffolds with minimum number of misassemblies clearly depict the efficiency and accuracy of CLA over other scaffolding tools. For example, in the *R*. *etli* genome of the simulated dataset, CLA could combine all 30 contigs into just two scaffolds whereas MeDuSa, Bambus2, SSPACE with extension, SSPACE without extension, SOPRA, SOAPdenovo2 and SGA generated 10, 23, 24, 28, 30, 24 and 30 scaffolds, respectively. CLA’s performance was consistent for all the genomes under comparison except in *H*. *pylori* where the number of misassemblies slightly increased to 7. In *C*. *jejuni*, 33 contigs were combined by CLA into 6 scaffolds without any misassemblies. In *B*. *quintana*, *C*. *crescentus* and *S*. Typhi, only 2 inversions were found within scaffolds from CLA (S4 Table). Numbers of misassemblies performed by CLA were relatively fewer when compared with MeDuSa even in the real dataset (Table 4).

**Table 3:** Comparative statistics of CLA and Scaffolding tools generated using simulated dataset.

	Genome*	Tool	# scaffolds	# misassemblies within scaffolds	# misassembled Scaffolds	Genome fraction
1.	***B*. *Quintana*** #contigs: **47** #misassemblies in input contigs: **0**	CLA	3	2	1	99.68
		MeDuSa	16	11	7	98.751
		Bambus2	32	3	1	98.639
		SSPACE (no extension)	41	1	1	98.763
		SSPACE (extension)	38	1	1	98.822
		SOPRA	43	1	1	98.751
		SOAPdenovo2	35	4	3	98.704
		SGA	45	0	0	98.743
2.	***C*. *jejuni*** #contigs: **33** #misassemblies in input contigs: **0**	CLA	6	0	0	99.728
		MeDuSa	10	18	1	98.756
		Bambus2	22	4	2	98.736
		SSPACE (no extension)	28	0	0	98.781
		SSPACE (extension)	24	2	2	98.829
		SOPRA	32	0	0	98.737
		SOAPdenovo2	28	3	1	98.792
		SGA	32	0	0	98.737
3.	***C*. *crescentus*** #contigs: **49** #misassemblies in input contigs: 0	CLA	4	2	1	99.901
		MeDuSa	12	24	7	99.356
		Bambus2	31	13	3	99.332
		SSPACE (no extension)	44	0	0	99.347
		SSPACE (extension)	38	3	3	99.363
		SOPRA	46	0	0	99.34
		SOAPdenovo2	46	0	0	99.34
		SGA	46	0	0	99.34
4.	***H*. *influenzae*** #contigs: **43** #misassemblies in input contigs: **1**	CLA	5	2	1	98.963
		MeDuSa	6	16	2	98.18
		Bambus2	31	6	3	98.158
		SSPACE (no extension)	38	2	2	98.003
		SSPACE (extension)	35	2	2	98.033
		SOPRA	26	1	1	98.156
		SOAPdenovo2	40	1	1	98.144
		SGA	27	1	1	98.156
5.	***H*. *pylori*** #contigs: **46** #misassemblies in input contigs: **0**	CLA	2	7	2	99.672
		MeDuSa	7	15	3	99.137
		Bambus2	25	10	6	99.103
		SSPACE (no extension)	39	0	0	99.101
		SSPACE (extension)	36	0	0	99.151
		SOPRA	42	0	0	99.092
		SOAPdenovo2	37	1	1	99.105
		SGA	44	0	0	99.078
6.	***R*. *etli*** #contigs: **30** #misassemblies in input contigs: **0**	CLA	2	2	1	99.811
		MeDuSa	10	13	1	99.204
		Bambus2	23	1	1	99.071
		SSPACE (no extension)	28	0	0	99.211
		SSPACE (extension)	24	2	2	99.225
		SOPRA	30	0	0	99.204
		SOAPdenovo2	24	4	1	99.215
		SGA	30	0	0	99.204
7.	***S*. Typhi** #contigs: **67** #misassemblies in input contigs: **0**	CLA	7	2	1	99.149
		MeDuSa	3	30	1	98.695
		Bambus2	47	11	6	98.666
		SSPACE (no extension)	51	1	1	98.679
		SSPACE (extension)	53	0	0	98.717
		SOPRA	63	0	0	98.647
		SOAPdenovo2	56	4	3	98.657
		SGA	66	0	0	98.644
8.	***T*. *pallidum*** #contigs: **22** #misassemblies in input contigs: **0**	CLA	3	3	2	99.124
		MeDuSa	1	13	1	98.765
		Bambus2	16	2	1	98.571
		SSPACE (no extension)	13	1	1	98.755
		SSPACE (extension)	14	2	1	98.865
		SOPRA	18	1	1	98.759
		SOAPdenovo2	14	2	2	98.747
		SGA	21	0	0	98.731

*All the genomes were simulated with a read length of 100bp and insert size of 400bp.

^#^ Number of

**Table 4:** Comparative statistics of CLA and Scaffolding tools generated using real dataset.

	Genome*	Tool	# scaffolds	# misassemblies within scaffolds	# misassembled scaffolds	Genome fraction
1.	***C*. *jejuni*** #contigs: **72** Read length: **100** Insert: **300** #misassemblies in input contigs: **7**	CLA	19	9	5	90.551
		MeDuSa	10	33	1	90.764
		Bambus2	45	16	9	90.692
		SSPACE (no extension)	50	10	8	90.589
		SSPACE (extension)	51	11	7	90.612
		SOPRA	44	9	6	90.685
		SOAPdenovo2	52	9	6	90.626
		SGA	54	9	5	90.613
2.	***E*. *coli*** #contigs: **252** Read length: **151** Insert: **300** #misassemblies in input contigs: **98**	CLA	67	165	37	92.31
		MeDuSa	74	223	11	92.047
		Bambus2	174	139	54	91.998
		SSPACE (no extension)	241	99	60	91.967
		SSPACE (extension)	249	104	63	92.132
		SOPRA	212	117	56	91.995
		SOAPdenovo2	206	103	56	91.935
		SGA	233	100	56	91.948
3.	***H*. *influenzae*** #contigs: **50** Read length: **100** Insert: **200** #misassemblies in input contigs: **25**	CLA	6	29	4	88.984
		MeDuSa	12	45	4	87.571
		Bambus2	43	25	10	87.562
		SSPACE (no extension)	32	26	11	87.533
		SSPACE (extension)	33	28	11	87.604
		SOPRA	24	27	6	88.184
		SOAPdenovo2	29	28	9	87.595
		SGA	34	28	4	88.148
4.	***M*. *tuberculosis*** #contigs: **241** Read length: **101** Insert: **300** #misassemblies in input contigs: **6**	CLA	44	10	5	98.52
		MeDuSa	9	93	3	98.549
		Bambus2	139	59	24	98.324
		SSPACE (no extension)	153	11	10	98.432
		SSPACE (extension)	144	14	11	98.534
		SOPRA	119	10	8	98.455
		SOAPdenovo2	108	12	12	98.365
		SGA	139	9	9	98.388
5.	***S*. Typhi** #contigs: **119** Read length:**100** Insert: **200** #misassemblies in input contigs: **2**	CLA	40	5	3	98.771
		MeDuSa	22	47	6	98.796
		Bambus2	80	19	10	98.771
		SSPACE (no extension)	85	6	5	98.803
		SSPACE (extension)	91	2	2	98.855
		SOPRA	80	8	4	98.712
		SOAPdenovo2	89	3	3	98.709
		SGA	119	2	2	98.717

* Real genome data used;

^#^ Number of

Though MeDuSa generated a lesser number of scaffolds in a few cases of both the real and simulated datasets, the number of misassemblies was significantly higher in all the organisms. While misassemblies in case of SSPACE, SOPRA and SOAPdenovo2 were more or less similar to that of CLA, these tools gave a higher number of scaffolds. Considering the trade-off between number of scaffolds generated and the number of misassemblies, CLA showed the best performance with least number of scaffolds having the minimum misassemblies and the highest genome fraction recovered.

### CLA versus combination of reference based and scaffolding tools

CLA being able to perform ordering followed by scaffolding, its performance was also compared by ordering already generated scaffolds. Ragout was used to order scaffolds from SSPACE with extension, SOPRA, SOAPdenovo2 and SGA and the final results were compared to that of CLA. The comparative statistics are listed out in Table 5.

**Table 5:** Comparative statistics of CLA and Ragout ordering of scaffolds using simulated dataset.

	Genome*	Tool	# of contigs and scaffolds in the input file	# misassemblies in chromosome assembly	Unassigned contigs from final order >1kb	Largest contig unassigned	Genome fraction
**1.**	***B*. *quintana*** #misassemblies in input contigs: **0**	CLA	47	2	0	850	99.68
		SSPACE_ext/Ragout	38	15	2	1155	99.242
		SOPRA/Ragout	43	7	6	3347	98.296
		SOAPdenovo2/Ragout	35	9	3	11040	98.644
		SGA/Ragout	45	2	0	739	99.153
**2.**	***C*. *jejuni*** #misassemblies in input contigs: **0**	CLA	33	4	0	425	99.728
		SSPACE_ext/Ragout	24	5	5	15071	97.389
		SOPRA/Ragout	32	5	4	4566	98.807
		SOAPdenovo2/Ragout	28	4	3	1954	99.094
		SGA/Ragout	32	3	4	4566	98.807
**3.**	***C*. *crescentus*** #misassemblies in input contigs: **0**	CLA	49	2	0	793	99.901
		SSPACE_ext/Ragout	38	32	0	371	99.386
		SOPRA/Ragout	46	8	0	371	99.896
		SOAPdenovo2/Ragout	46	6	0	371	99.923
		SGA/Ragout	46	6	0	371	99.923
**4.**	***H*. *influenzae*** #misassemblies in input contigs: **1**	CLA	43	6	0	606	98.963
		SSPACE_ext/Ragout	35	11	1	1088	98.136
		SOPRA/Ragout	26	11	0	572	99.013
		SOAPdenovo2/Ragout	40	12	0	375	98.517
		SGA/Ragout	27	3	1	2461	98.247
**5.**	***H*. *pylori*** #misassemblies in input contigs: **0**	CLA	46	7	0	960	99.672
		SSPACE_ext/Ragout	36	12	1	6406	98.657
		SOPRA/Ragout	42	4	1	5487	98.965
		SOAPdenovo2/Ragout	37	6	1	10376	98.746
		SGA/Ragout	44	4	1	5487	98.97
**6.**	***R*. *etli*** #misassemblies in input contigs: **0**	CLA	30	3	0	601	99.756
		SSPACE_ext/Ragout	24	8	5	3972720	89.983
		SOPRA/Ragout	30	5	1	1052	99.406
		SOAPdenovo2/Ragout	24	7	4	3831	99.102
		SGA/Ragout	30	4	1	1052	99.501
**7.**	***S*. Typhi** #misassemblies in input contigs: **0**	CLA	67	8	0	520	99.141
		SSPACE_ext/Ragout	53	61	1	5127	98.625
		SOPRA/Ragout	63	9	0	519	99.236
		SOAPdenovo2/Ragout	56	32	0	283	99.104
		SGA/Ragout	66	54	3	3835	99.142
**8.**	***T*. *pallidum*** #misassemblies in input contigs: **0**	CLA	22	5	0	980	99.124
		SSPACE_ext/Ragout	14	9	1	1696	98.711
		SOPRA/Ragout	18	6	1	1617	99.535
		SOAPdenovo2/Ragout	14	10	0	244	99.513
		SGA/Ragout	21	8	1	1617	99.537

*All the genomes were simulated with a read length of 100bp and insert size of 400bp.

^#^ Number of

CLA performed better with the least number of misassemblies in the majority of cases like *B*. *quintana*, *C*. *crescentus*, *R*. *etli*, *S*. Typhi and *T*. *pallidum*. Ragout and SGA together generated the least number of misassemblies in *C*. *jejuni*, *H*. *influenzae* and *H*. *pylori* followed by CLA. Amongst the combinations used, Ragout and SGA gave good results similar to that of CLA while Ragout and SSPACE with extension did not seem to be an ideal and consistent combination.

### Additional features of CLA

#### Handling intra-contig repeating segments

Small repetitive sequences which form only part of a contig also create problems during assembly. Some of these elements consist of insertion sequences (IS) and transposable elements. CLA could split and precisely place certain segments of contigs which comprised of IS and transposable elements and displayed connections at different places, given sufficient availability of paired-end information. Such segments within the contigs are extracted based on the read alignment positions and are placed according to their connections in the map-file ([b] in S1 Fig). CLA in simulated data of *S*. Typhi placed a 500-600bp intra-contig repeat at about 24 places. This segment was found to be a transposable element that was present at about 26 different positions in the original genome of *S*. Typhi TY2. The positioning of these small repeats according to CLA and their original position in the reference are mentioned in S5 Table. The performance of CLA in terms of placing small repeats appeared to vary and was dependent on the sequencing quality and coverage depth. Therefore, CLA appears to be an efficient tool in handling even small repetitive elements in comparison to other tools which could not address these issues.

#### Contigs unrelated to the chromosomal assembly

Possible contamination of DNA samples during sequencing and inaccurate de-multiplexing of read data may lead to contamination of the reads and potentially resulting in un-related contigs. Such contigs might cause significant problems during downstream analysis. In other cases, plasmids which are not a part of chromosomal sequences also pose problems in achieving accurate genome assembly. Given this, CLA was observed to filter out such contigs and separate them from the main chromosomal assembly. Contigs with BLASTn identity (against reference) of less than 5% and with no paired-end link information are tagged as unrelated contigs. Such contigs were effectively excluded out by CLA in real datasets of *E*. *coli*, *H*. *influenzae*, *H*. *pylori*, *Mycobacterium tuberculosis* and *S*. Typhi. In *S*. Typhi, one of the contigs tagged as unrelated was found to be a plasmid contig thus preventing false scaffolding with the chromosomal contigs. To examine the performance of CLA in the detection of possible contamination, a few random contigs from other genomes were introduced into simulated data. CLA could detect these contigs as contamination from a different source and correctly labelled them as possibly unrelated contigs. Contigs tagged as unrelated could be cross-checked by the user for its use in the final assembly.

#### Information about probable misassemblies

Another advantage of using CLA is that the user is provided with additional files containing information on the efficiency of read-pair mapping and the extent of possible misassembling. The same can be useful for advanced users in order to further improve their assembly. CLA flags this information by vending a log file to the user.

## Discussion

Genome assembly could be a challenging task especially for prokaryotes. This is mainly due to the plasticity of prokaryotic genomes as dictated by discrete evolutionary events and bottlenecks that shape adaptation dynamics and lifestyles of bacterial organisms in different ecosystems [1]. Consequently, prokaryotic genomes are often replete with signatures reminiscent of various genetic rearrangements occurring due to frequent insertion, deletion and substitution events as well as enriched with multiple homopolymeric tracts (arising out of replication errors), repeat motifs of different composition and lengths, insertion sequences, prophages and genomic islands etc. [27]. All these plastic regions pose serious difficulties in assembling a genome mainly because of sequence redundancy that they bring in the form of multiple alleles, palindromes, inverted repeats and tandem duplications. Most of the available tools either rely on reference to order contigs at the whole genome level, or scaffold them based on the read data [7, 9, 10, 14, 16, 18]. While using a reference genome could result in omission/exclusion of certain strain specific elements [10], the scaffolding methods struggle to resolve repetitive regions and limit ordering within the scaffolds [20]. Given these practical difficulties, we developed CLA, which combines the benefits of individual approaches to minimize errors while generating a draft genome. CLA uses a reference at the beginning to create a preliminary sort order which then undergoes extensive validation based on paired-end read information to resolve repetitive elements and re-sorting of the contigs. The sorted contigs are only scaffolded based on the available read-pair information. Although ordering is tried to be attained at the whole genome level, contigs are linked into scaffolds only when supported by paired-end information indicating their connectivity. Hence, it is easier for the users to fill in the information between the scaffolds using further downstream processing in order to achieve a complete genome.

The existing reference based tools though efficient in sorting the contigs at the whole genome level, were observed to remove those contigs from the final chromosome assembly which failed to properly align with the reference (Tables 1 and 2). These excluded contigs may lead to loss of significant genome information. For example, BLAST analysis of such excluded contigs from the *H*. *influenzae* genome identified several genes encoding metabolic functions, which were otherwise discarded. On the other hand, the scaffolding of contigs is performed only based on paired-end information [14, 18, 19] or in the case of MeDuSa using multiple reference genomes [24]. In the case of final assembly/genome obtained with multiple scaffolds, ordering is limited within the scaffolds and also repetitive regions with their misleading connections sometimes lead to intra-scaffold misassemblies. From our validation study, it was observed that CLA could address all these issues better than the compared tools (Tables 1–5). Since CLA utilizes both reference and paired-end information, it performed better in retaining maximum number of contigs in the final output without compromising on accuracy. It also utilizes read-pair information to place some of the repetitive elements. The overall performance of CLA was found to be much better than the existing reference based ordering tools as well as scaffolding tools with a minimum number of misassemblies. In all our case studies and comparative analyses, CLA was seen to be misplacing contigs in just two cases: 1) firstly, when there was insufficient paired-end information, 2) secondly, when two contigs had same flanking contig connections at both ends leading to their misjoining within the scaffold. To avoid such scenarios, CLA lists out contigs with probable swapping, in a log file to alert the users of probable misassemblies within scaffolds.

The performance with the real and simulated datasets pointed out the capability of CLA in not only handling data from monomorphic bacteria such as *S*. Typhi but also highly diverse ones as *H*. *pylori*. The higher abundance of transposases in bacteria and important biological roles proposed for them in previous studies underlines the need to handle them carefully during genome assembly [28]. CLA was observed to be efficient in this aspect and was able to resolve 24 out of 26 transposable elements in *S*. Typhi.

Though manual curation is inevitable for completing a genome, CLA leaves less scope for manual intervention and also provides all the required blueprints to complete further re-construction of the genome. Therefore, we believe that in the light of existing difficulties regarding the genome assembly, CLA would be a significant step forward in improving the genome assembly pipeline with a user friendly approach and efficient data usage.

## Materials and Methods

### Real and simulated datasets

Eight complete bacterial genomes with varied genome characteristics were downloaded from NCBI. The paired reads were simulated for each of the genomes with the help of GemSIM (v.1.6) [29] using its Illumina error model. The genome characteristics of these genomes along with their accession numbers are provided in S1 Table. The 100 bases long read-pairs were simulated with an insert length of 400(±20) along with genome coverage of 100X. The reads were filtered using NGS QC Toolkit (v.2.3) [30] to remove bad quality reads.

These filtered reads were then assembled into contigs using Velvet *de novo* assembler (1.2.08) with an optimal k-mer chosen with the help of VelvetOptimiser (2.2.5) (http://bioinformatics.net.au/software.velvetoptimiser.shtml). The assembled contigs were used as input for validating CLA with other tools using QUAST. For the real dataset, five paired-read datasets with different organismal background from SRA data were considered. Information about these datasets is provided in the S2 Table. Filtering followed by the assembly of reads was carried out in a similar manner as described above.

### Pipeline of CLA

The tool was designed and developed to run in the following stages:

#### Sort order creation

**Fig 1:**
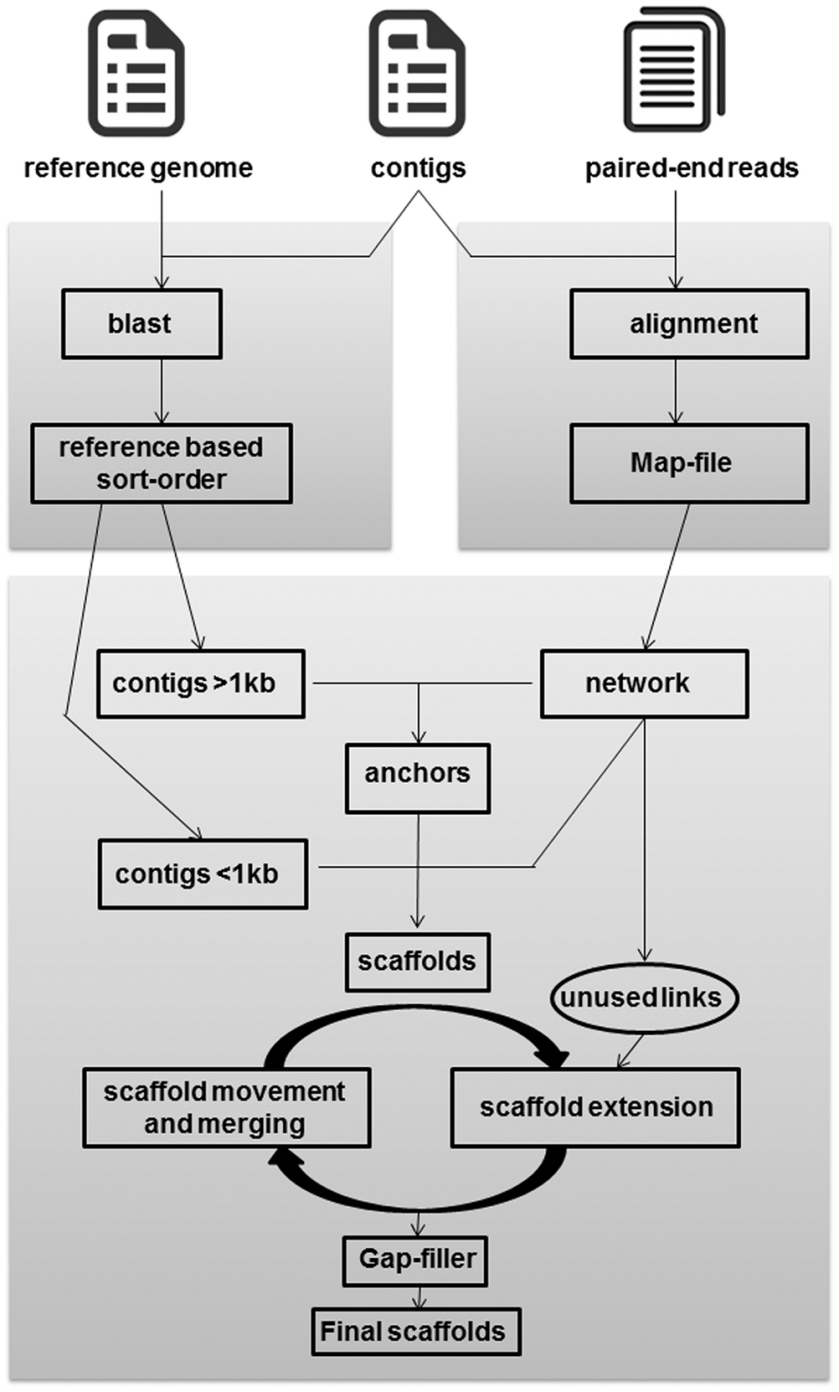
Schematic overview of CLA pipeline. The schema of CLA pipeline is divided into three stages. In the first stage, a reference based order is derived followed by the second stage where connections between the individual contigs are extracted based on alignment information. The final stage makes use of the information from the first two stages to decide the final order followed by scaffolding and gap filling.

The detailed schematic of CLA is explained in Fig 1. Contigs, paired-end sequence reads and a suitable reference genome sequence (preferably of the same species as the genome being assembled) serve as input for the tool. The process starts with the exclusion of contigs that are less than 200bp. A preliminary sort order is created after alignment of the contigs with the reference genome by using BLASTn. Contigs with a BLASTn best hit of less than a threshold identity value (for contigs of size <1kb: 50% identity; size>1-10kb: 25% identity; size >10kb: 10% identity) are placed at the end of the order. The individual contigs are arranged according to the sort order with consequent reverse complementation of contigs wherever required in accordance with BLASTn output.

#### Extracting the connections

**Fig 2:**
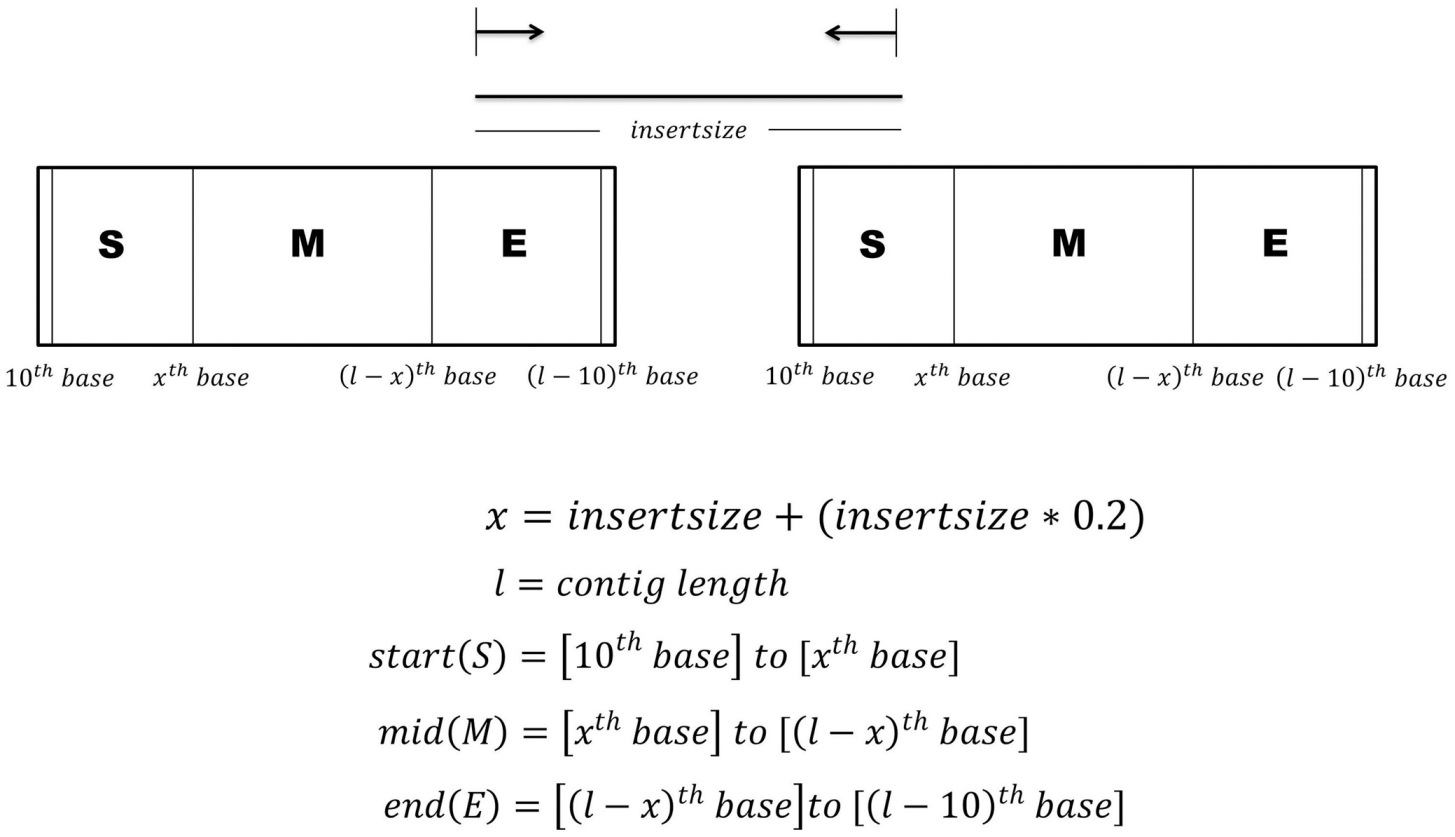
Defining the start and end of a contig based on the insert size. Connectivity between two consecutive contigs is decided by the paired reads from a single insert. To find such connections between contigs, each contig is theoretically divided into start, mid and end regions based on the insert size.

The paired reads are then mapped to the sorted contigs (obtained as above) using BWA [31] and their information regarding the alignment position and orientation is extracted. Each of the contigs is theoretically divided into start, mid and end regions as depicted in Fig 2 wherein the sequence read having its pair in any other contig constitutes a potential link. The information of all possible links (formed by the read pairs) at the start, mid and end regions of each of the contigs is then tabulated in a map file. Since the read pairs are derived from a single insert, these links strongly suggest the proximity or contiguity of these two contigs in the real genome. For example, as depicted in Fig 2, if two contigs are in proximity then the reads aligned at the end of one contig would have their linking pairs aligned at the start of the following contig. The map file contains four columns representing the contig ID, start, end and mid region of the contigs, respectively. To avoid any erroneous assembly at the extreme ends of the contigs, the first and last ten bases from the start and the end of each of the contigs are not considered for calculating the links. A minimum of 10 read pair links are considered to calculate the proximity of two contigs in the genome (valid connection between contigs). Therefore, the map file generation is instrumental in validation of the reference sorted contigs and also helps in resolving some of the issues such as repeats, duplications and inversions. Some examples regarding the resolutions of repetitive regions and possible duplications are detailed in S1 Fig.

#### Sort-order validation

Contigs that have less than 5% BLASTn identity against reference (as inferred from the earlier BLASTn output) and lacking suitable connections in the map-file are flagged as un-related and are removed from the downstream analysis. These might represent contigs formed due to contaminating reads or sometimes due to plasmid contigs as observed from the results of the real datasets. A network file is then created from the map file, where start/end of a contig is represented as vertex/node and their respective connections as edges. Contigs that are less than 1kb in size are initially excluded from the sort order. The tool then scans the map file to validate the reference based sort order while looking for connections between the neighboring contigs (contigs >1kb). In cases where there is no direct connection (no links connecting the end of the former and start of the later contig) between two consecutive contigs from the reference based sort order, the tool then looks for any intermediary contigs linking these two contigs based on the link information from the map file. Each path is a representation of one or more contigs and a shortest path (defined by one or more contigs connecting to other contigs based on the link information gleaned from the map file) was found using the Floyd Warshall algorithm [32]. Only paths that include contigs greater than 1kb in size are considered at this stage. The connecting contig is either copied or moved from its position based on the connections in the map file. All the contigs at the end of this stage are referred to as anchors.

#### Connecting the anchors

The link information of the excluded contigs of size less than 1kb were then used to connect the defined anchors using the Floyd Warshall algorithm as discussed in the previous section. All such connected anchors were then merged into scaffolds after placing ‘Ns’ between them. To prevent any false positive placements, the sort-order was scanned at every step based on the number of connections from the map file.

#### Ordering the scaffolds

An iterative process of scaffold merging and extension is performed by the CLA based on the unused connections and leftover contigs from the network file. After a search for inter scaffold connections, if two scaffolds are found to be connected, the one smaller in size is moved and oriented in accordance with the larger scaffold. Once merged, the map-file is scanned for any further unused connections at the ends of the newly extended scaffolds. Merging is again performed and the process is repeated until either all the connections are exhausted or no proper connections could be found. A final sort order is then created and the contigs are merged into scaffolds followed by their ordering, all in accordance with the new sort order. The gaps between the scaffolds are then closed by using GapFiller [33]. Even though the order is defined, all the contigs are not merged into a single pseudogenome and are left at the stage of scaffolds because of lack of valid connections available from the map file. Thus, inter-scaffold gaps indicate the existence of gaps at the level of the sequencing data itself. CLA uses the FASTX toolkit (http://hannonlab.cshl.edu/fastx_toolkit/) for easy handling and manipulation of the intermediate files.

## Supporting Information

S1 Fig Pictorial representation of various scenarios where map-file can be used to resolve repetitive contigs. (a) A normal case scenario where a repetitive contig 38 is placed at two different positions based on its connections from the map-file. (b) An example of an intra-contig repeating segment, where mid-region of contig 20 is connecting two contigs—contig 9 and contig 35. (c) Example of a tandem repeat, where the whole contig 95 has connections at both start and end pointing to another contig 82.
(TIF)

S1 Table Genome characteristics and information of the strains utilized for simulation. Table with the accession numbers of the strains and their genomic characteristics that were used for the simulated dataset
(PDF)

S2 Table Information about strains under real dataset. Table with the accession numbers of the strains and reference genomes used for the study
(PDF)

S3 Table Misassembly details of CLA and reference based ordering tools in simulated dataset. Table listing out number of relocations, translocations and inversions which amounted to the total number of misassemblies
(PDF)

S4 Table Misassembly details of CLA and Scaffolding tools in simulated dataset. Table listing out number of relocations, translocations and inversions which amounted to the total number of misassemblies
(PDF)

S5 Table BLAST output of intra-contig repeat from CLA result in simulated *S*. Typhi Ty2 data against the original Ty2 genome. The positions tabulated depict that a ~600bp intra-contig repeat is present at 26 different locations in the original genome
(PDF)
