# Supplementary figures and images for: Contig-Layout-Authenticator (CLA): A Combinatorial Approach to Ordering and Scaffolding of Bacterial Contigs for Comparative Genomics and Molecular Epidemiology
Source: PLoS One. 2016 Jun 1;11(6):e0155459. doi: 10.1371/journal.pone.0155459 (PMC4889084; doi:10.1371/journal.pone.0155459)

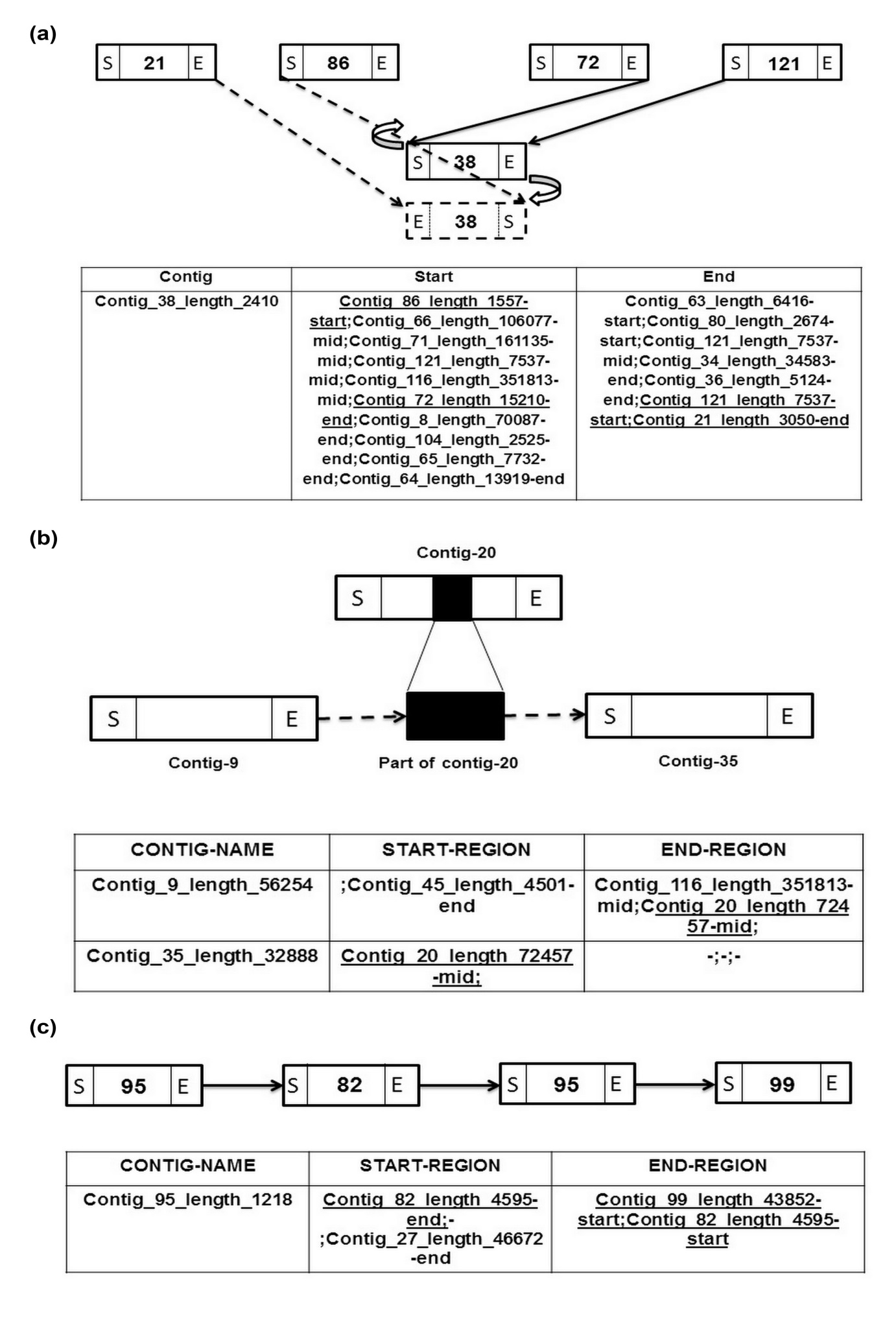

Supplement: S1 Fig — (a) A normal case scenario where a repetitive contig 38 is placed at two different positions based on its connections from the map-file. (b) An example of an intra-contig repeating segment, where mid-region of contig 20 is connecting two contigs—contig 9 and contig 35. (c) Example of a tandem repeat, where the whole contig 95 has connections at both start and end pointing to another contig 82. (TIF) [file pone.0155459.s001.tif]
