# Supplementary material for: Contig-Layout-Authenticator (CLA): A Combinatorial Approach to Ordering and Scaffolding of Bacterial Contigs for Comparative Genomics and Molecular Epidemiology
Source: PLoS One. 2016 Jun 1;11(6):e0155459. doi: 10.1371/journal.pone.0155459 (PMC4889084; doi:10.1371/journal.pone.0155459)
Supplement: S1 Table — Table with the accession numbers of the strains and their genomic characteristics that were used for the simulated dataset (PDF) [file pone.0155459.s002.pdf]

S1 Table: Genome characteristics and information of the strains utilized for simulation
Table with the accession numbers of the strains and their genomic characteristics that were used for the simulated dataset

Sl. No.	Bacteria used for simulating paired-end information	GenBank ID	(G+C)%	Feature	Genome size in bp##	reference genomes** (GenBank IDs)
1	***Bartonella quintana* str. Toulouse**	BX897700.1	38.8	Clonal	1581384	CP003784.1<br>BX897699.1*<br>CP001562.1*
2	***Campylobacter jejuni* NCTC 11168**	AL111168.1	30.55	Highly recombining	1641481	HG428754.1<br>CP003871.3*<br>CP000025.1*
3	***Caulobacter crescentus* CB15**	AE005673.1	67.21	High G+C %	4016947	CP001340.1<br>CP002008.1*<br>CP000927.1*
4	***Haemophilus influenzae* Rd KW20**	L42023.1	38.15	Highly recombining	1830023	FQ312006.1<br>CP005967.1*<br>CP002277.1*
5	***Helicobacter pylori* J99**	AE001439.1	39.19	Highly recombining	1643831	CP001217.1<br>CP000012.1*<br>AE000511.1*
6	***Rhizobium etli* CFN 42**	CP000133.1	61.27	Clonal, high G+C %	4381608	CP005950.1<br>CP001074.1*<br>CP006986.1*
7	***Salmonella* Typhi Ty2**	AE014613.1	52.05	Clonal	4791950	CP003278.1<br>CP007559.1*<br>AL513382.1*
8	***Treponema pallidum* str. Fribourg-Blanc**	CP003902.1	52.78	Highly clonal	1140253	CP003115.1<br>CP002375.1*<br>CP002374.1*

** Reference strains used for their respective simulated data while validating the tools
*Additional reference genomes used for running MeDuSa and Ragout
##Genome size of the original genome that is used for simulation
