# Supplementary material for: Contig-Layout-Authenticator (CLA): A Combinatorial Approach to Ordering and Scaffolding of Bacterial Contigs for Comparative Genomics and Molecular Epidemiology
Source: PLoS One. 2016 Jun 1;11(6):e0155459. doi: 10.1371/journal.pone.0155459 (PMC4889084; doi:10.1371/journal.pone.0155459)
Supplement: S2 Table — Table with the accession numbers of the strains and reference genomes used for the study (PDF) [file pone.0155459.s003.pdf]

**S2 Table: Information about strains under real dataset**

Table with the accession numbers of the strains and reference genomes used for the study

| Sl. No. | Bacteria in the real dataset | SRA accession numbers | Reference genomes** (GenBank IDs) |
|---|---|---|---|
| 1. | ***Campylobacter jejuni*** | SRR1999732 | CP006006.1<br>HG428754.1*<br>CP003871.3*<br>CP000025.1* |
| 2. | ***Escherichia coli*** | SRR1788079 | CP006636.1<br>HG941718.1*<br>CP006784.1*<br>CP000819.1* |
| 3. | ***Haemophilus influenzae*** | DRR015774 | CP005967.1<br>FQ312006.1*<br>CP007471.1*<br>CP002277.1* |
| 4. | ***Mycobacterium tuberculosis*** | ERR845304 | CP001664.1<br>HE663067.1*<br>AP012340.1*<br>CP009480.1* |
| 5. | ***Salmonella enterica*** | ERR351254 | AE006468.1<br>CP012151.1*<br>AL513382.1*<br>CP012091.1* |

**Reference strains used for CLA, reference based ordering tools and for generating QUAST statistics

*Additional reference strains and reference genomes used for running MeDuSa and Ragout
