# Supplementary material for: Contig-Layout-Authenticator (CLA): A Combinatorial Approach to Ordering and Scaffolding of Bacterial Contigs for Comparative Genomics and Molecular Epidemiology
Source: PLoS One. 2016 Jun 1;11(6):e0155459. doi: 10.1371/journal.pone.0155459 (PMC4889084; doi:10.1371/journal.pone.0155459)
Supplement: S3 Table — Table listing out number of relocations, translocations and inversions which amounted to the total number of misassemblies (PDF) [file pone.0155459.s004.pdf]

S3 Table: Misassembly details of CLA and reference based ordering tools in simulated dataset

Table listing out number of relocations, translocations and inversions which amounted to the total number of misassemblies

	**Genome***	**Tool**	**# relocations**	**# translocations**	**# inversions**
1.	***B. quintana*** #contigs:**47** #total repeat positions: **19** #misassemblies in input contigs: **0**	CLA	0	0	2
		Ragout	5	0	0
		ABACAS	12	0	0
		MCM	26	0	0
		CONTIGuator	4	0	0
2.	***C. jejuni*** #contigs: **33** #total repeat positions: **12** #misassemblies in input contigs: **0**	CLA	4	0	0
		Ragout	4	0	1
		ABACAS	9	0	0
		MCM	21	0	0
		CONTIGuator	8	0	0
3.	***C. crescentus*** #contigs:**49** #total repeat positions: **28** #misassemblies in input contigs: **0**	CLA	0	0	2
		Ragout	8	0	0
		ABACAS	11	0	0
		MCM	18	0	0
		CONTIGuator	4	0	0
4.	***H. influenza*** #contigs: **43** #total repeat positions: **19** #misassemblies in input contigs: **1**	CLA	6	0	0
		Ragout	12	0	0
		ABACAS	15	0	0
		MCM	18	0	4
		CONTIGuator	9	0	2
5.	***H. pylori*** #contigs: **46** #total repeat positions: **20** #misassemblies in input contigs: **0**	CLA	5	0	2
		Ragout	2	0	2
		ABACAS	15	0	2
		MCM	21	0	2
		CONTIGuator	7	0	1
6.	***R. etli*** #contigs: **30** #total repeat positions: **21** #misassemblies in input contigs: **0**	CLA	3	0	0
		Ragout	5	0	0
		ABACAS	5	0	0
		MCM	23	0	1
		CONTIGuator	10	0	0
7.	***S.* Typhi** #contigs: **67** #total repeat positions: **24** #misassemblies in input contigs: **0**	CLA	8	0	0
		Ragout	55	0	0
		ABACAS	13	0	0
		MCM	32	0	0
		CONTIGuator	13	0	0
8.	***T. pallidum*** #contigs: **22** #total repeat positions: **17** #misassemblies in input contigs: **0**	CLA	5	0	0
		Ragout	6	0	1
		ABACAS	10	0	0
		MCM	14	0	1
		CONTIGuator	6	0	0

*All the genomes were simulated with a read length of 100bp and insert size of 400bp. # No. of
