# Supplementary material for: Contig-Layout-Authenticator (CLA): A Combinatorial Approach to Ordering and Scaffolding of Bacterial Contigs for Comparative Genomics and Molecular Epidemiology
Source: PLoS One. 2016 Jun 1;11(6):e0155459. doi: 10.1371/journal.pone.0155459 (PMC4889084; doi:10.1371/journal.pone.0155459)
Supplement: S4 Table — Table listing out number of relocations, translocations and inversions which amounted to the total number of misassemblies (PDF) [file pone.0155459.s005.pdf]

S4 Table: Misassembly details of CLA and Scaffolding tools in simulated dataset
Table listing out number of relocations, translocations and inversions which amounted to the total number of misassemblies

	Genome*	Tool	# relocations	# translocations	# inversions
1.	***B. quintana*** #contigs:**47** #misassemblies in input contigs: **0**	CLA	0	0	2
		MeDuSa	10	0	1
		Bambus2	3	0	0
		SSPACE (no extension)	1	0	0
		SSPACE (extension)	1	0	0
		SOPRA	1	0	0
		SOAPdenovo2	4	0	0
		SGA	0	0	0
2.	***C. jejuni*** #contigs:**33** #misassemblies in input contigs: **0**	CLA	0	0	0
		MeDuSa	18	0	0
		Bambus2	4	0	0
		SSPACE (no extension)	0	0	0
		SSPACE (extension)	2	0	0
		SOPRA	0	0	0
		SOAPdenovo2	3	0	0
		SGA	0	0	0
3.	***C. crescentus*** #contigs:**49** #misassemblies in input contigs: **0**	CLA	0	0	2
		MeDuSa	22	0	2
		Bambus2	13	0	0
		SSPACE (no extension)	0	0	0
		SSPACE (extension)	3	0	0
		SOPRA	0	0	0
		SOAPdenovo2	0	0	0
		SGA	0	0	0
4.	***H. influenzae*** #contigs:**43** #misassemblies in input contigs: **1**	CLA	2	0	0
		MeDuSa	16	0	0
		Bambus2	6	0	0
		SSPACE (no extension)	2	0	0
		SSPACE (extension)	2	0	0
		SOPRA	1	0	0
		SOAPdenovo2	1	0	0
		SGA	1	0	0
**5.**	***H. pylori*** #contigs:**45** #misassemblies in input contigs: **0**	CLA	5	0	2
		MeDuSa	15	0	0
		Bambus2	9	0	1
		SSPACE (no extension)	0	0	0
		SSPACE (extension)	0	0	0
		SOPRA	0	0	0
		SOAPdenovo2	1	0	0
		SGA	0	0	0
6.	***R. etli*** #contigs:**46** #misassemblies in input contigs: **0**	CLA	2	0	0
		MeDuSa	13	0	0
		Bambus2	1	0	0
		SSPACE (no extension)	0	0	0
		SSPACE (extension)	2	0	0
		SOPRA	0	0	0
		SOAPdenovo2	4	0	0
		SGA	0	0	0

7.	***S.* Typhi**<br><br>#contigs:**67**<br>#misassemblies in input contigs: **0**	CLA	0	0	2
		MeDuSa	30	0	0
		Bambus2	11	0	00
		SSPACE (no extension)	1	0	0
		SSPACE (extension)	0	0	0
		SOPRA	0	0	0
		SOAPdenovo2	4	0	0
		SGA	0	0	0
8.	***T. pallidum***<br><br>#contigs:**22**<br>#misassemblies in input contigs: **0**	CLA	3	0	0
		MeDuSa	13	0	0
		Bambus2	2	0	0
		SSPACE (no extension)	1	0	0
		SSPACE (extension)	2	0	0
		SOPRA	1	0	0
		SOAPdenovo2	2	0	0
		SGA	0	0	0

*All the genomes were simulated with a read length of 100bp and insert size of 400bp. # No. of
