# Supplementary material for: Contig-Layout-Authenticator (CLA): A Combinatorial Approach to Ordering and Scaffolding of Bacterial Contigs for Comparative Genomics and Molecular Epidemiology
Source: PLoS One. 2016 Jun 1;11(6):e0155459. doi: 10.1371/journal.pone.0155459 (PMC4889084; doi:10.1371/journal.pone.0155459)
Supplement: S5 Table — The positions tabulated depict that a ~600bp intra-contig repeat is present at 26 different locations in the original genome (PDF) [file pone.0155459.s006.pdf]

S5 Table: BLAST output of intra-contig repeat from CLA result in simulated *S.* Typhi Ty2 data against the original Ty2 genome

The positions tabulated depict that a ~600bp intra-contig repeat is present at 26 different locations in the original genome.

	**Intra contig repeat segment**	**Reference**	**%identity**	**Start position in reference**	**End position in reference**
1	Contig_48_length_3835[1557-2173]	gi|29140506|gb|AE014613.1|	100	3536792	3536176
2	Contig_48_length_3835[1557-2173]	gi|29140506|gb|AE014613.1|	93.98	1898116	1898729
3	Contig_48_length_3835[1557-2173]	gi|29140506|gb|AE014613.1|	94.11	2323287	2323896
4	Contig_48_length_3835[1557-2173]	gi|29140506|gb|AE014613.1|	94.11	2534267	2534876
5	Contig_48_length_3835[1557-2173]	gi|29140506|gb|AE014613.1|	94.1	528845	528237
6	Contig_48_length_3835[1557-2173]	gi|29140506|gb|AE014613.1|	94.09	1802022	1802630
7	Contig_48_length_3835[1557-2173]	gi|29140506|gb|AE014613.1|	94.1	2999401	3000009
8	Contig_48_length_3835[1557-2173]	gi|29140506|gb|AE014613.1|	94.1	3108592	3107984
9	Contig_48_length_3835[1557-2173]	gi|29140506|gb|AE014613.1|	94.08	1189885	1189279
10	Contig_48_length_3835[1557-2173]	gi|29140506|gb|AE014613.1|	93.69	1745293	1745908
11	Contig_48_length_3835[1557-2173]	gi|29140506|gb|AE014613.1|	93.93	3077484	3078093
12	Contig_48_length_3835[1557-2173]	gi|29140506|gb|AE014613.1|	93.93	674093	673485
13	Contig_48_length_3835[1557-2173]	gi|29140506|gb|AE014613.1|	93.93	1221320	1220712
14	Contig_48_length_3835[1557-2173]	gi|29140506|gb|AE014613.1|	94.07	1265695	1265090
15	Contig_48_length_3835[1557-2173]	gi|29140506|gb|AE014613.1|	93.93	1451127	1450519
16	Contig_48_length_3835[1557-2173]	gi|29140506|gb|AE014613.1|	93.93	1647569	1646961
17	Contig_48_length_3835[1557-2173]	gi|29140506|gb|AE014613.1|	93.93	1894909	1895517
18	Contig_48_length_3835[1557-2173]	gi|29140506|gb|AE014613.1|	93.93	2116469	2117077
19	Contig_48_length_3835[1557-2173]	gi|29140506|gb|AE014613.1|	93.93	3012263	3012871
20	Contig_48_length_3835[1557-2173]	gi|29140506|gb|AE014613.1|	93.93	4604899	4605507
21	Contig_48_length_3835[1557-2173]	gi|29140506|gb|AE014613.1|	93.78	1613193	1613802
22	Contig_48_length_3835[1557-2173]	gi|29140506|gb|AE014613.1|	93.77	271634	271026
23	Contig_48_length_3835[1557-2173]	gi|29140506|gb|AE014613.1|	93.77	3085076	3085684
24	Contig_48_length_3835[1557-2173]	gi|29140506|gb|AE014613.1|	93.62	279969	280578
25	Contig_48_length_3835[1557-2173]	gi|29140506|gb|AE014613.1|	93.44	2473149	2473757
26	Contig_48_length_3835[1557-2173]	gi|29140506|gb|AE014613.1|	93.44	465283	464676
